# Insights on Microplastic Contamination from Municipal and Textile Industry Effluents and Their Removal Using a Cellulose-Based Approach

**DOI:** 10.3390/polym16192803

**Published:** 2024-10-03

**Authors:** Solange Magalhães, Daniel Paciência, João M. M. Rodrigues, Björn Lindman, Luís Alves, Bruno Medronho, Maria da Graça Rasteiro

**Affiliations:** 1 University of Coimbra, CERES, Department of Chemical Engineering, 3030-790 Coimbra, Portugal; solangemagalhaes@eq.uc.pt (S.M.); danielpacienciarodrigues@hotmail.com (D.P.); 2 CICECO—Aveiro Institute of Materials, Department of Chemistry, University of Aveiro, 3810-193 Aveiro, Portugal; jrodrigues@ua.pt; 3 Physical Chemistry, University of Lund, P.O. Box 124, SE-221 00 Lund, Sweden; bjorn.lindman@fkem1.lu.se; 4 Coimbra Chemistry Center (CQC), Department of Chemistry, University of Coimbra, Rua Larga, 3004-535 Coimbra, Portugal; 5 MED—Mediterranean Institute for Agriculture, Environment and Development, CHANGE—Global Change and Sustainability Institute, Faculty of Sciences and Technology, University of Algarve, Campus de Gambelas, Ed. 8, 8005-139 Faro, Portugal; bfmedronho@ualg.pt; 6 Surface and Colloid Engineering, FSCN Research Centre, Mid Sweden University, Holmgatan 10, SE-851 70 Sundsvall, Sweden

**Keywords:** microplastics, textile and municipal effluents, wastewater treatment plant, polyethylene terephthalate (PET)

## Abstract

The rampant use of plastics, with the potential to degrade into insidious microplastics (MPs), poses a significant threat by contaminating aquatic environments. In the present study, we delved into the analysis of effluents from textile industries, a recognized major source of MPs contamination. Data were further discussed and compared with a municipal wastewater treatment plant (WWTP) effluent. All effluent samples were collected at the final stage of treatment in their respective WWTP. Laser diffraction spectroscopy was used to evaluate MP dimensions, while optical and fluorescence microscopies were used for morphology analysis and the identification of predominant plastic types, respectively. Electrophoresis was employed to unravel the prevalence of negative surface charge on these plastic microparticles. The analysis revealed that polyethylene terephthalate (PET) and polyamide were the dominant compounds in textile effluents, with PET being predominant in municipal WWTP effluents. Surprisingly, despite the municipal WWTP exhibiting higher efficiency in MP removal (ca. 71% compared to ca. 55% in textile industries), it contributed more to overall pollution. A novel bio-based flocculant, a cationic cellulose derivative derived from wood wastes, was developed as a proof-of-concept for MP flocculation. The novel derivatives were found to efficiently flocculate PET MPs, thus allowing their facile removal from aqueous media, and reducing the threat of MP contamination from effluents discharged from WWTPs.

## 1. Introduction

**Figure 1:**
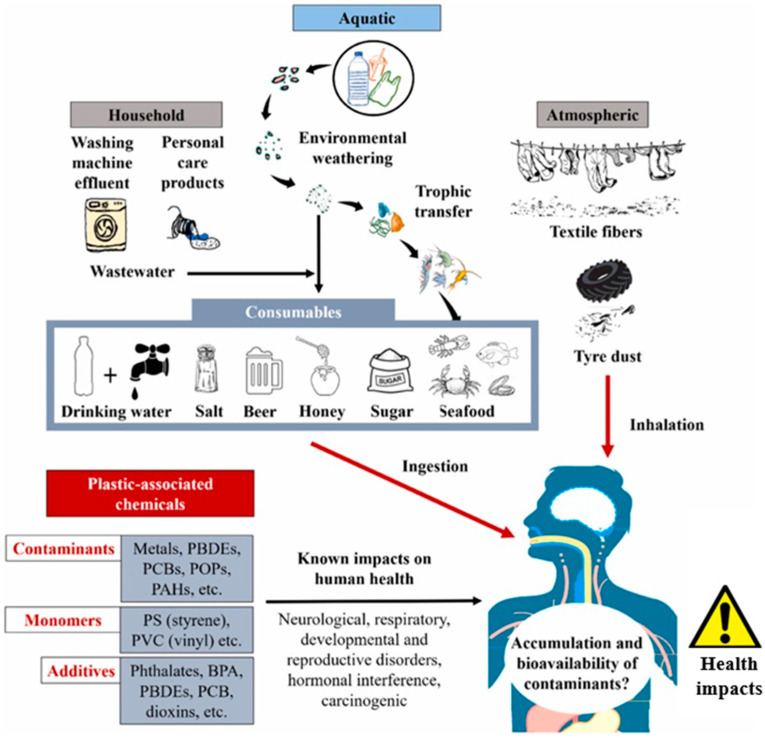
Sources and potential impact of MPs in ecosystems. Taken from reference [5] with permission of Elsevier.

According to a 2018 report by Plastics Europe, global plastic production has grown exponentially, reaching nearly 350 million tons in 2017 [1]. The world is virtually flooded with plastic waste, with an estimated 6 billion tons circulating globally. Moreover, plastics contain over 10,000 chemicals, many of which are harmful, including carcinogens and endocrine disruptors [2]. Scientists have already warned that if the rapid increase in plastic production continues, plastics could outnumber fish in the sea by 2050. While these staggering numbers have raised global awareness, the lack of knowledge regarding smaller plastic fragments, such as microplastics (MPs), makes future projections uncertain. MPs have garnered significant attention worldwide due to their potentially harmful effects on ecosystems. These particles are routinely detected across a wide range of environments, including freshwater, seawater, the atmosphere, sediments, soils, effluents, and even food [3,4]. Due to their small size (less than 5 mm) and limited biodegradability, MPs can be ingested by various organisms, entering the marine food chain. This poses a twofold threat: first, to the environment and its ecosystems, and second, to human health (Figure 1).

In addition to their inherent risks, their association with toxic additives, such as heavy metals, pharmaceutical drugs, pesticides, additives used during plastic processing, and other persistent organic pollutants, amplifies the danger of their presence in the environment [5]. These contaminants have been linked to various human illnesses and diseases, including obesity, diabetes, cancer, endocrine disturbance, cardiovascular issues, and reproductive problems [6].

Among all of the MP forms identified in the environment, fibres emerge as especially intriguing. These fibres, frequently resulting from extensive textile washing processes, predominantly trace back to widely utilized synthetic textile polymers, such as polyester, polyamide, acrylic, and polyolefin polymers, notorious for their limited biodegradability [7,8]. The manufacturing process of synthetic fibres starts with the preparation of the filaments, usually obtained by extrusion, where the melted polymers are forced through small orifices and then solidify [8]. These filaments manifest in diverse forms, either as continuous structures known as yarns or as discontinuous structures, referred to as staple fibres. These filaments are subjected to a thermal drawing process, enhancing their mechanical properties, including tenacity. During this process, molecules like polyethylene terephthalate (PET) undergo a dynamic reorientation in the fibre direction and crystallize. Therefore, the crystallinity of the fibre depends on the applied draw ratio [9]. Drawn filaments are then combined and further processed in different ways to form yarns with specific characteristics, culminating in a diverse array of textile materials and products [7,10,11].

It has been described in the literature that MPs can enter the environment through textile washing processes [12,13,14]. During washing, both the mechanical action and chemical actions of detergents affect the reactive groups of fibres [15], weakening their structure. Such progressive damage in the molecular polymer chains can lead to a substantial reduction in the degree of polymerization (DP), consequently breaking the fibre into small particles (MPs) [10]. It has been shown that in the case of cotton and silk fibres, when wet, tumescence facilitates fibrils release through physical forces [16]. Prolonged contact between the textile article and water can induce intense fibre degradation. It has also been demonstrated that higher water-volume washing cycles result in increased MP release [17]. Although information regarding the behaviour of synthetic fibres in aqueous mediums is scarce and usually not mentioned in the literature, they are expected to be less affected by tumescence phenomena due to their prevalent hydrophobic nature [8]. Besides water interaction, temperature is also expected to play an important role in fabric degradation. In cases where the washing temperature exceeds 70 °C, irreversible structural transformations in the synthetic material can be observed, leading to fibre degradation, even after only a few washing cycles [18]. In brief, several factors significantly influence the structure of the textile fibres, potentially enhancing their release into the environment. Therefore, it is crucial to study the contribution of different effluents, such as those from the textile industry, which is often considered a significant source of MP pollution, and compare them to municipal effluents [12]. Textile industries often demand substantial water usage during production, dyeing, and finishing processes, resulting in the discharge of large volumes of residual water after completing the production cycle. Some industries have an in-house WWTP, which pre-treats the effluents before directing them to the municipal collector. Municipal WWTPs have long been recognized as a major contributor to MP pollution in aquatic environments. These facilities receive various types of MPs, including microfibers, microbeads, fragments, pellets, and foam. These particles primarily originate from everyday products such as facial cleansers, scrubs, cosmetics, sunscreen, nail polish, hair dye, eyeshadow, shower gels, and toothpaste. Additionally, MPs are formed by the fragmentation of larger plastic waste into smaller particles through mechanical, biological, chemical, and photo-oxidative degradation [19]. For example, the Plum Island WWTP in the U.S. receives between 8 billion and 20 billion MPs daily in its influent. In comparison, two other WWTPs, Rifle Range and Center Street, handle between 1 billion and 4 billion MPs per day. Meanwhile, the Ryaverket WWTP in Sweden processes approximately 201.2 kg of MPs in its influent each day [20]. The accurate detection and efficient removal of MPs from wastewater remain difficult due to the lack of standardized protocols and effective removal technologies. Despite progress in the characterization and quantification of MPs, their polydisperse nature and resistance to degradation, coupled with the design limitations of conventional WWTPs, enable MPs to evade treatment processes and enter the environment [19]. The primary objective of this work relies on understanding which effluent sources (i.e., industrial textile effluent or municipal effluent) release a higher concentration of MPs. Recently, we randomly selected Portuguese industries and compared their effluents regarding the MP load [21,22]. Surprisingly, the textile industry was not identified as the most polluting industry among the different sectors considered (i.e., textile, paint, resin, PVC and pharmaceutical). This work extends our previous research by providing a quantitative and qualitative analysis of the MP contributions from two different textile effluents, comparing them to a municipal WWTP in the central area of Portugal. The MP extraction followed the procedure developed in our laboratory [21], with some adjustments for the municipal effluent, considering its specific characteristics. On the other hand, the quantification and identification of MPs were performed by Fourier Transform Infrared Spectroscopy (FTIR), Laser Diffraction Spectroscopy (LDS), and optical and fluorescence microscopies. Currently, the most widely used method for determining the chemical composition of MPs is FTIR spectrophotometry [1]. This technique is preferred for its directness, reliability, and non-destructive nature. Different MPs generate distinct infrared spectra with specific band patterns, enabling precise identification [23]. Fluorescence microscopy offers a rapid screening method for detecting and identifying MPs by capturing fluorescent emissions from samples excited by specific wavelengths, with appropriate filter cubes or lasers aiding in the process [24]. Nile Red or Nile Blue are commonly used to stain MPs. These dyes bind to the hydrophobic surfaces of plastic particles, allowing them to fluoresce under the microscope; depending on the MP type, the fluorescence properties change allowing for MP identification.

While literature presents studies evaluating the content of MPs in the laundry washing of polyester textiles [17] and their release from garments and home textiles during washing, drying, and wearing [25], as well as the content of MPs in municipal WWTPs [26], no study, to the best of our knowledge, has compared the contribution of industrial and municipal WWTP effluents quantitatively and qualitatively. Beyond identifying and quantifying MPs in the different effluents, the development of efficient strategies to remove them is of major importance. Among the different strategies, flocculation is a widely used process in industrial applications, where the flocculant adsorbs onto colloidal particles, and the polymer re-conforms to reach an energy-minimized state. As these individual entities interact, larger aggregates (called flocs) are formed. Flocs settle faster, facilitating the removal of contaminants in settlement tanks. In the literature, Fe_2_(SO_4_)_3_ and other flocculants commonly used in secondary sedimentation, such as polyacrylamides, have been suggested to remove MPs by aggregating them into flocs (source: *Industrial Water Treatment Process Technology*). MPs trapped in unstable flocs may not be effectively removed, leading to their redistribution and eventual escape during clarification [27]. A stronger interaction, such as ionic interactions between MPs and flocculant agents, could potentially improve removal efficiency. In this work, we present a proof-of-concept using an innovative cationic cellulose-based flocculant, and compare its performance to a standard cationic polyacrylamide, demonstrating its effectiveness in removing model MPs from aqueous media via flocculation. The bioflocculant was produced applying a bio-based approach, using cellulose as raw material. It was prepared using a two-step procedure, in which first the cellulose ring was opened using sodium periodate, to form a cellulose dialdehyde (DAC) [28] intermediate compound. The second step involved the addition of cationic moieties to the polymer through the reaction of DAC with Giard T reagent (Figure S1). The preparation of water-soluble cationic celluloses directly from cellulose, without the oxidation step and the formation of DAC, involves the dissolution of cellulose in a suitable reaction media [29]. Since the dissolution of cellulose is a challenging process, using a two-step cationization approach eliminates the need for a dissolution step, simplifying the preparation of cationic cellulose derivatives.

## 2. Materials and Methods

### 2.1. Experimental Materials

Effluents from the in-house WWTP of two Portuguese textile industries and a municipal WWTP from the Coimbra region (centre of Portugal) were collected and analysed before and after the effluents being submitted to the WWTP treatment, not only to evaluate the removal efficiency of each treatment plant but also to access the potential MP contamination caused by each treated effluent. Two samples from each effluent were collected one hour apart, using a clean 5-litre glass container, and mixed before analysis to ensure that any unexpected fluctuations in composition were accounted for, which might have occurred if only a single sample had been taken. Glass fibre filters with a pore size of 1–2 μm and ultrapure water (UPW) were employed for all extraction and characterization steps. Hydrogen peroxide aqueous solution (30 wt%) was obtained from Greendet, Lda, Coimbra, Portugal. Potassium hydroxide (KOH) was purchased from LabKem, Barcelona, Spain. HCl (37%) was purchased from Honeywell, Lisboa, Portugal. Pyrene (98%) was obtained from Acros Organics from, Shanghai, China. Prior to use, all of the reagents were filtered through 0.45 µm syringe PTFE filters to minimize potential contamination by external particles.

A glass vacuum filtration unit (FiltresRS par Jean-Pierre D., Paris, France) and glass fibre filters, with a pore size of 1–2 μm, were used for the filtration of all of the samples. The filtration unit was covered with a watch glass to minimize potential contamination. The glass fibre filters were burned at 250 °C for 30 min before use.

Eucalyptus wood waste pulp (containing 83 wt% cellulose, 6 wt% hemicellulose, 5 wt% lignin, and 1.4 wt% extractives and ashes) was used as the starting cellulose material for the preparation of the bio-based flocculant. Sodium hydroxide pellets and glacial acetic acid were obtained from the VWR company (Porto, Portugal), while isopropanol was purchased from Labsolve (Bangkok, Thailand). Sodium periodate and (carboxymethyl)trimethylammonium chloride hydrazide (Girard’s reagent T) were acquired from Sigma-Aldrich (Algés, Portugal). 2-propanol was supplied by VWR, while sodium periodate, lithium chloride, hydroxylammonium chloride, and sodium acetate were obtained as p.a. grade from Sigma-Aldrich (Algés, Portugal). All chemicals were used as received without further purification and distilled water was used throughout the work.

Polyethylene (PE) and polyethylene terephthalate (PET) pellets (virgin plastic), as well as plastic from a water bottle, were processed to produce “model” microplastics for the nuclear magnetic resonance (NMR) studies and the flocculation proof-of-concept. In brief, the plastics were ground in a mill (knife mill, supplied by Thomas Scientific, Swedesboro, NJ, USA) and passed through a 200 µm sieve.

### 2.2. Sample Preparation and Extraction of the Microplastics

The extraction and analysis of MPs involve protocols that often require different preparation steps and long digestion times. In our previous work, we have developed a standard approach that relies on an initial alkaline treatment to eliminate organic matter, employing a 10% KOH (aq.) treatment during 12 h at 50 °C [21,30]. Following digestion in an alkaline medium, the samples were filtered through a glass fibre filter. Subsequently, the collected MPs were cleaned with H_2_O_2_ (10 mL) and ethanol, to remove any microorganisms from their surfaces. It is important to highlight that each extraction protocol must be customized based on the properties of the effluent (e.g., the amount of organic and inorganic compounds and/or other contaminants) to be studied [21]. In the case of the municipal effluent, a higher amount of hydrogen peroxide (30 mL) was used due to the large concentration of algae present. Two replicates of each extraction were performed, and strict quality control procedures were adopted for sample preparation and MP extraction. To ensure the integrity of the procedures, all glassware was meticulously cleaned before use, and the sample containers (beakers, filtration apparatus, etc.) were covered with a clock glass to minimize airborne sample contamination. Laboratory benches were also always cleaned before use.

### 2.3. Characterization and Quantification of Microplastics

The size and shape of the MPs present in the effluents (before and after treatment) were analysed following the method developed in our previous work [21]. Laser diffraction spectroscopy (LDS) assays were conducted to study the size distribution of the MPs (Mastersizer 2000 from Malvern Instruments, Malvern, UK, equipped with the Hydro 2000 module). The pump speed was set to 1500 rpm, which corresponds to an average shear rate of 334 s^−1^. The effluent samples were added to the dispersion unit of the equipment until obscuration reached an adequate level (i.e., 6–7% obscuration for optimal signal-to-noise quality). The results were processed using the Mastersizer 2000 software. A Zetasizer NanoZS (ZN 3500, Malvern Instruments, UK) was used to determine the charge density of MPs by measuring the zeta potential of the effluent samples via the electrophoretic light scattering technique. In brief, the samples were gently transferred to a folded capillary zeta cell and visually checked for the presence of air bubbles. After equilibration at 25 °C, each sample was scanned 3 times, with acquisition runs automatically set by the software. The results were processed using the Zetasizer Nano Software 7.13 (Malvern Instruments, Malvern, UK).

The quantification of the MPs was conducted by gravimetry. The filters were weighed before the filtration process (after being burned at 250 °C for 30 min) and reweighed after filtering 100 mL of effluent. In this later step, filters were dried in an oven at 105 °C for 8 h. The MP load in each effluent was estimated based on the mass difference of the filter before and after filtration.

The size and shape of MPs were also analysed after filtration using an optical Olympus BH-2 KPA microscope (Olympus Optical Co., Ltd., Tokyo, Japan) equipped with a high-resolution CCD colour camera (ColorView III, Olympus Optical Co., Ltd., Tokyo, Japan).

Additionally, microparticles of PE and PET were studied by solid-state ^13^C cross-polarization (CP) magic-angle spinning (MAS) NMR in a Bruker Avance III 400 spectrometer (Billerica, MA, USA) (9.4T), featuring 3.7 μs ^1^H 90° pulses, 3500 ms contact time, spinning rates of 12 kHz, and 5 s recycle delays.

### 2.4. Identification of Microplastics

Following the strategy previously reported [21], the identification of the main polymer types present in the isolated MPs was performed by Fourier-transform infrared spectroscopy (FTIR), and also optical and fluorescence microscopies. The FTIR spectra were obtained using an ATR-FTIR (Perkin-Elmer FT-IR spectrometer, Hopkinton, MA, USA) with a universal ATR sampling accessory, spanning from 500 cm^−1^ to 4000 cm^−1^, with a resolution of 4 cm^−1^ and applying 128 scans. The obtained spectra of the MPs in the effluents before and after the WWTP treatment were compared with an existing FTIR library (Aldrich Collection FT-IR Spectra, Boston, MA, USA). Similarly, the spectra of the raw material of the most common MP particles found (polyethylene, PE; polypropylene, PP; polyethylene terephthalate, PET; and nylon) were obtained from pellets of the mentioned materials. Before acquiring the FTIR spectra, the filters containing the MPs were observed in the optical microscope to identify the zones with a higher concentration of microparticles. These areas were marked to facilitate filter placement in the ATR.

Fluorescence microscopy was also employed to characterize the type of polymers present in the isolated MPs. Samples were stained with a fluorescent dye, and the colour of the particles was analysed after excitation and fluorescence emission. Pyrene was selected as the fluorescent probe due to its excellent sensitivity to surface-modified carboxyl particles [31]. Although common plastics, such as PE and polypropylene, do not naturally contain carboxylic groups, these groups could be introduced into the referred polymers by oxidation and UV exposure, leading to polymer chain degradation and the formation of smaller particles [32,33]. In brief, 200 μL of the pyrene stock solution (1 mg mL^−1^) was diluted into 20 mL of ultra-pure milli-Q water and poured into the filtration funnel. To ensure effective staining, the solution was allowed to contact the glass fibre filter and retained MPs for ca. 15 min. After this period, the excess solution was removed by vacuum filtration and the filter disc was placed in a petri dish. Samples were imaged at room temperature in a fluorescence microscope (Olympus BX51M, Tokyo, Japan), equipped with a 100 × objective lens, a filter set type U-MNU2 (360–370 nm excitation and 400 nm dichromatic mirror), and a UV-mercury lamp (100 W Ushio Olympus, Tokyo, Japan). Images were digitized using a video camera (Olympus digital camera DP70, Tokyo, Japan) and analysed with an image processor (Olympus DP Controller 2.1.1.176, Olympus DP Manager 2.1.1.158, Tokyo, Japan).

### 2.5. Synthesis of Bio-Based Flocculant

Cellulose cationization was performed using a two-step procedure, following the method described by Grenda et al. [34]: First, the formation of dialdehyde cellulose (DAC) was conducted, followed by the cationization with Girard’s T reagent (CDAC). For this, Eucalyptus wood pulp (composed of ca. 85 wt% of the cellulose, 14 wt% of the hemicellulose, and <0.1 wt% of the lignin) was dispersed in water at 4 wt% and the suspension was then treated with a mixture of 300 mL of distilled water, 7.2 g of LiCl, and 8.2 g of NaIO_4_ at 75 °C in the absence of light. After a 3 h reaction, a highly oxidised material was produced. Then, the oxidised cellulose was filtered and washed with distilled water. The degree of substitution (DS) of aldehyde groups was determined by titration with sodium hydroxide, based on the oxime reaction between the aldehyde groups and hydroxylamine hydrochloride (Figure S2) [35]. In the subsequent step, the undried DAC (DS = 1.63) was dispersed in acidified water at a consistency of 5 wt%, and a specific molar ratio of Girard’s reagent T to aldehyde groups was added. The cationization was performed at 70 °C for 1 h. The resulting cationic samples were vacuum filtered through a filter paper with a pore size of 11 μm and washed with a mixture of isopropanol/water (9:1 in volume) until the conductivity of the filtrate was constant, minimizing the loss of solubilised cellulose. The nitrogen content (wt%) of the cationic cellulose was determined by elemental analysis (EA 1108 CHNS-O analyser from Fisons) and used to determine the degree of cationic substitution, assuming that all detected nitrogen comes from the grafted quaternary ammonium cations and the Eucalyptus wood waste pulp is composed exclusively by cellulose. The native cellulose, and obtained DAC and CDAC were characterized by FTIR as shown in Figure S3. The charge density was estimated using a potentiometric titration method [36]. The obtained cationic cellulose derivative (coded as 48B), which was further used as bioflocculant for MP removal, exhibited a final cationic DS of 1.02, a charge density of 3.26 mmol·g^−1^, and a zeta potential value of +51 mV. More details about the bioflocculant preparation and characterisation are given in the Supplementary Materials.

### 2.6. Zeta Potential

A Zetasizer NanoZS from Malvern Instruments was used to measure the zeta potential of the cationic cellulose polymer in solution and PET MPs in two different pHs (pH 7.0 and 3.0). Each sample was measured 5 times with 15 sub-runs for each measurement. The zeta potential was measured at 25 °C with an equilibration time of 60 s.

### 2.7. Flocculation Tests

The evolution of the size distribution of PET microparticles in aqueous suspension was monitored in a Mastersizer 2000 device (from Malvern Instruments) equipped with the Hydro 2000 module. Before the measurements, approximately 300 mg of MPs were suspended in water (600 mL) using a pump speed of 1000 rpm. After a 5 min dispersion, the bio-based flocculant was added at a concentration of 0.001 wt% (i.e., 6 mg of bioflocculant per flocculation assay) keeping the speed at 1000 rpm. This concentration was selected based on previous work [4]. After 10 min, the stirring speed was reduced to 500 rpm to allow the formation of flocs. The flocculation was allowed to continue for 60 min while monitoring the particle size evolution (d_0.5_) as a function of the flocculation time.

## 3. Results and Discussion

Understanding the extent of MP contamination, particularly the contributions from municipal and industrial WWTPs, has been the driving force behind the present study. As mentioned before, textile effluents are commonly considered significant sources of MP pollution in ecosystems [25,37]. Thus, this work focussed on the analysis and comparison of effluents from two different textile industries with an effluent from a municipal WWTP from the centre of Portugal. It is well-documented that effluents from the textile industry usually contain nylon and PE polymers [38,39], while municipal WWTP effluents often comprise a heterogenous mixture with high organic and biological fractions [39,40]. Table 1 presents the zeta potential and pH values of the studied effluents before the extraction procedure. It is important to note that two samples were collected for each type of effluent, both before applying the treatment and after the treatment (in the case of the textile industries, this refers to their in-house WWTP).

The first striking observation is that the effluent from the textile 1 industry exhibits a much higher pH than the other two effluents. This may be related to some specific processes used in the company, such as bleaching, where alkaline solvents and oxidizing agents are often employed. Compounds like sodium hydroxide or other strong bases are used to maintain a high pH, typically ranging between 12–12.5 [41]. In the case of textile 2 effluent, the company employs different wet processing routes, including dyeing, printing, and finishing, which may consume ca. 80% water and 20% chemicals, thus resulting in more neutral effluents [42].

**Figure 2:**
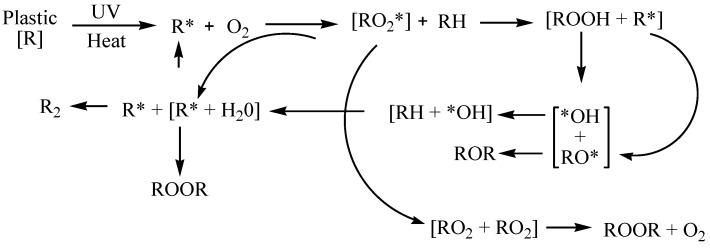
Polymer free radical chain reaction.

The average zeta potential of all effluents is negative, ranging between ca. –12 mV and –31 mV. In addition to various factors that can contribute to a negative zeta potential, it is important to highlight that microplastics (MPs) typically carry a negative charge. This characteristic significantly affects the surface charge of particles throughout the entire effluent. Considering the expected greater compositional complexity of the municipal effluent, it is reasonable to assume that the partial neutralization of highly charged particles may easily occur, leading to an overall lower zeta potential when compared to simpler textile effluents. The negative charge of MPs may result from various factors. The degradation of macroplastics occurs due to exposure to various biotic and abiotic-like stresses, including UV and thermal radiation, mechanical impacts, and the growth of microorganisms on the surface. At elevated temperatures, such as those encountered in industrial processes like extrusion and casting, degradation is induced through a thermo-oxidative reaction. This reaction initiates a chain reaction of polymer-free radicals, leading to a self-propagating oxidative process (Figure 2). The reaction continues until the external energy source is removed, or the oxygen supply is cut off, stopping the process. Alternatively, radicals may combine to form stable non-radical adducts, effectively terminating the reaction. However, such termination is rare due to the infrequent collision of radicals. Ultimately, the temperature at which this oxidative reaction occurs is determined by the specific thermal properties of the plastic material and the presence of oxygen [33,43,44].

Another mode of degradation is photo-oxidative degradation, which involves the breakdown of plastic upon exposure to light. Most plastics are susceptible to UV light due to the presence of photo-reactive groups known as chromophores. These groups readily absorb high-energy UV radiation, leading to the rupture of chemical bonds. The photo-oxidative degradation of plastic material occurs when photons of UV light, particularly in the UVB region (315–280 nm) of the electromagnetic spectrum, initiate the decomposition process through a free radical polymer chain reaction.

**Figure 3:**
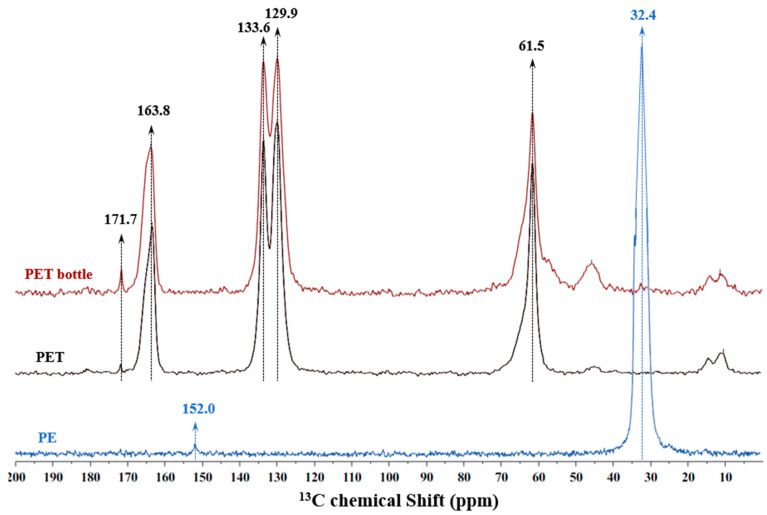
Solid-state CP/MAS ^13^C NMR of PE (blue line), PET (black line), and a PET bottle obtained from a commercial water bottle (red line).

Throughout these processes, in the presence of oxygen, surface oxidations are frequently observed on the plastic particles, resulting in the formation of carboxylic groups. To confirm this possibility, “model” MPs (PE and PET) were studied by solid-state NMR to assess the presence of different functional groups on the structure of the plastics (Figure 3).

The PE “model” MPs do not reveal the presence of relevant peaks in the chemical shift range of functional groups typical of material oxidation, such as ketones, esters, or carboxylic acids (165–185 ppm) [45]. This explains the low zeta potential observed for “model” MP suspensions at neutral to alkaline pH, ca. –12 mV, as the presence of carboxylic groups is quite low and was not observed in NMR experiments (below the detection/quantification limit of NMR). On the other hand, PET “model” MPs, both from PET pellets and water bottles, exhibited peaks at 163.8 and 171.7 ppm, which are assigned to the presence of esters (characteristic of PET), and carboxylic acids (resulting from PET structure degradation), respectively [45,46]. Another interesting observation is that the intensity of the peak at 171.7 ppm for the MPs produced from the water bottle is higher than that observed for the PET from pellets. This is certainly related to the higher degradation of the PET structure induced by the thermal treatment during the production of the water bottle, leading to the formation of a higher number of carboxylic groups. The NMR results are well aligned with the zeta potential values of PET MP pellets, which presented a value of ca. –45 mV at neutral pH.

**Figure 4:**
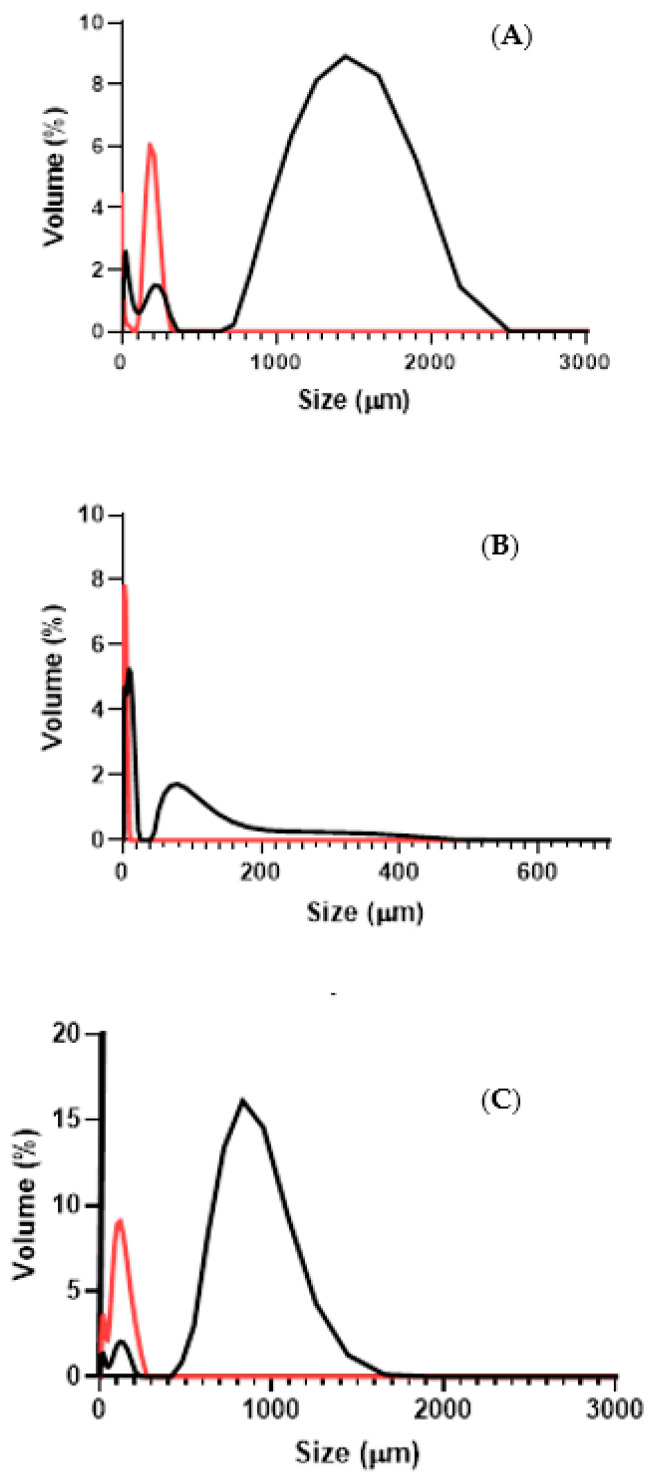
Size distribution of the textile and municipal effluents before treatment (black curves) and after treatment (red curves), measured by LDS. The effluents correspond (**A**) textile 1; (**B**) textile 2; and (**C**) municipal effluent.

The average particle size in the effluents was characterised for all of the samples, before and after the WWTP (Figure 4).

The textile 1 and municipal effluents are the ones that presented the larger particles in suspension. These are also the systems that exhibited the least negative zeta potential, which might contribute to some particle aggregation and formation of larger aggregates. Aggregation could also be responsible for the broader size distribution observed in these systems. On the other hand, due to the higher charge density of textile 2 effluent, particles are less prone to aggregate (electrostatic repulsion) and the average particle size is smaller and the size distribution is narrower. After the treatments, the average particle size is observed to substantially decrease in all effluents. We believe this reflects the satisfactory capacity of the WWTP systems to handle the larger particles and remove them by sedimentation and filtration. On the other hand, the fraction of smaller particles increases in all effluents after treatment, which might be a direct consequence of larger particle/aggregate destruction during such a procedure. Plastics are known to be susceptible to fragmentation as a result of abrasion and turbulence, which commonly occur in WWTPs [4].

Following the procedure described in Section 2.1, the MPs from the different effluents were also quantified, before and after the in-house treatments (Table 2). Although the removal efficiency of MPs is observed to be higher for the municipal WWTP, the number of MPs released is still much higher than the amount released from the textile industries. This is because WWTP effluents often contain not only domestic waste but also discharges from other sources. Additionally, the higher release of MPs from the municipal WWTP, even when considering only those from textiles, can be attributed to the massive and extensive clothes-washing cycles, which lead to the continuous release of MPs into wastewater. Also, the MPs released by clothes are usually microfibers, which can contribute to reducing the capacity of the municipal WWTP to trap the small fragments of fibres, due to their long and thin shape, explaining the larger quantity of MPs released by this WWTP compared to the textile industry effluents.

It is worth noticing that the paint and resin industries (which use acrylic polymers and PET) previously evaluated [21] still present a higher average release of MPs than the municipal WWTP and the textile industries analysed here.

**Figure 5:**
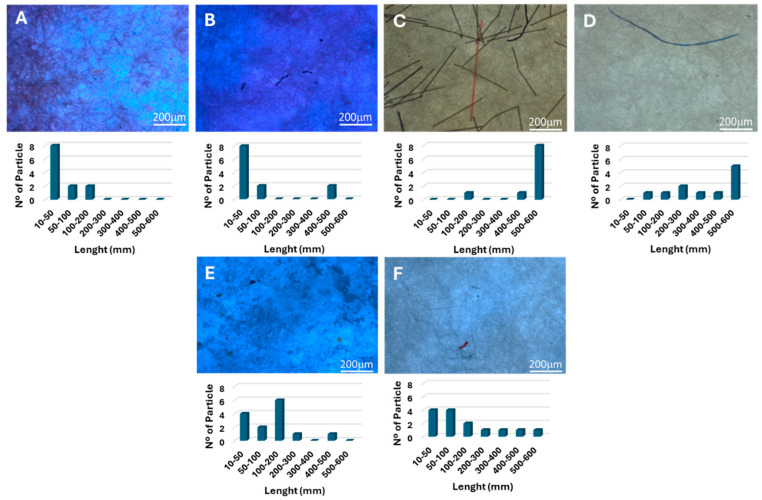
Optical micrographs and size distributions of the collected MPs before (**A**,**C**,**E**) and after (**B**,**D**,**F**) the WWTP treatment. Textile effluent 1 (**A**,**B**), textile effluent 2 (**C**,**D**), and municipal effluent (**E**,**F**).

As shown in Table 2, it is possible to observe that the effluent from the municipal WWTP has a greater load of MPs in both stages of collection (before and after WWTP treatment), with a higher percentage also being retained, although a considerable number of MPs is still released into the environment. It is worth mentioning that municipal WWTPs treat a significantly larger volume of water compared to other WWTPs; therefore, this factor should definitely contribute to the higher initial and released loads of MPs detected. Yarns from fabrics are often challenging to remove or flocculate in the WWTP process and, because of this, they can reach the water courses and increase contamination by MPs. As alluded to, fibres are expected to constitute most of the MPs present in the studied effluents, as their shape favours easier passage through the WWTP processes, including the filtration steps. It is thus not surprising that most of the MPs identified in the textile effluents were fibre-like, while in the case of the municipal WWTP effluent, elongated fibres and small fragments were detected (Figure 5).

The smaller MPs detected in this work may have originated either from the specific nature of the effluents tested and/or partial MP degradation during the cleaning procedure. The alkaline and oxidizing treatments used to clean and extract the MPs may further fractionate them [31,37]. Previously, we have observed that the “WWTP procedure” could affect the average particle size (Figure 4) [21]. The distributions in Figure 5 show similar tendencies although the distributions obtained from micrographs do not coincide exactly with Figure 4. Still, we can observe that the reported particle size tends to decrease upon “WWTP procedure”.

**Figure 6:**
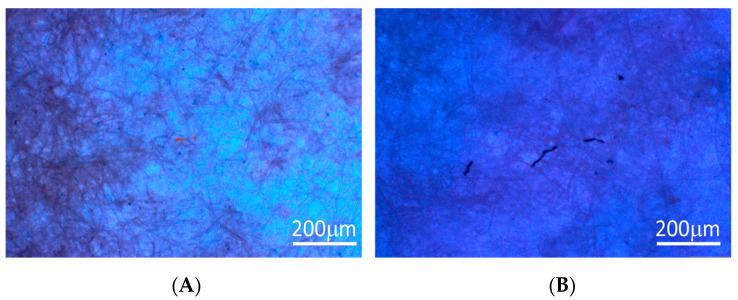
Optical micrographs of the collected MPs before (**A**) and after (**B**) for textile industry 1, as an example.

Additionally, to infer the eventual effect of our cleaning procedure on the MPs, such as a possible degradation of the surface and/or size changes of MPs, samples were analysed before and after the cleaning procedure. Figure 6 presents images of the textile 1 effluent before and after the sample cleaning procedure applied in this work.

From the images in Figure 6 and the histograms in Figure 5, it can be inferred that the shape and size of the MPs did not change remarkably, demonstrating that the cleaning process has low to no effect on the characteristics of the MPs present in the effluent.

**Figure 7:**
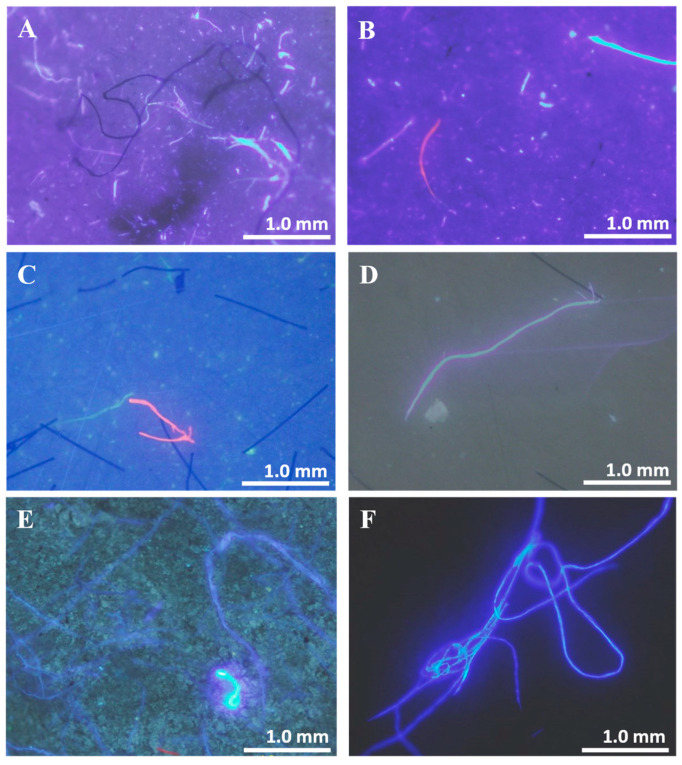
Fluorescence microscopy images of the MPs before (**A**,**C**,**E**) and after (**B**,**D**,**F**) the WWTP treatment; textile 1 effluent (**A**,**B**), textile 2 effluent (**C**,**D**) and municipal effluent (**E**,**F**). Samples were stained with pyrene (see the Section 2.1 for more details).

Additionally, fluorescence microscopy was performed not only to infer the shape and size of the MPs but mainly to assess the type of polymer present in the MPs (Figure 7).

Fluorescence microscopy is a useful method to identify MPs. The interaction of the MPs with a chromophore leads to the development of a certain colour for each MP, which is a function of the material properties, such as the dielectric constant [47]. Materials with different polarities have different dielectric constants (for example, polypropylene, PP: 2.2–2.5; high density polyethylene, HDPE: 2.3–2.4; low density polyethylene, LDPE: 2.2–2.35; polystyrene, PS: 2.4–3.1; and PET: >3). The spectral emission of the chromophore shifts to higher wavelengths, ranging from blue and green, for more hydrophobic plastics like PP and PE, to red when interacting with more polar plastics, such as nylon or PET [47,48].

From Figure 7, it is possible to identify the presence of PET (particles with pink colour), as well as polyamide (particles with red colour), (Figure 7B,C) in the effluents, which are in accordance with literature [21,47]. In the case of the effluents from the municipal WWTP, the presence of PET (particles with pink colour) can be inferred. Regardless of the effluent, a high number of PET microparticles are always observed, supported by the fact that this is the most used thermoplastic polymer in the world, and it Is also widely used in the textile industry under the trade name “polyester”, representing nowadays ca. 30% of the fibres used for clothing [49]. Nevertheless, other potential sources or factors may affect the prevalence of the MPS, such as other industrial activities, atmospheric deposition or the degradation of larger plastic items [50].

**Figure 8:**
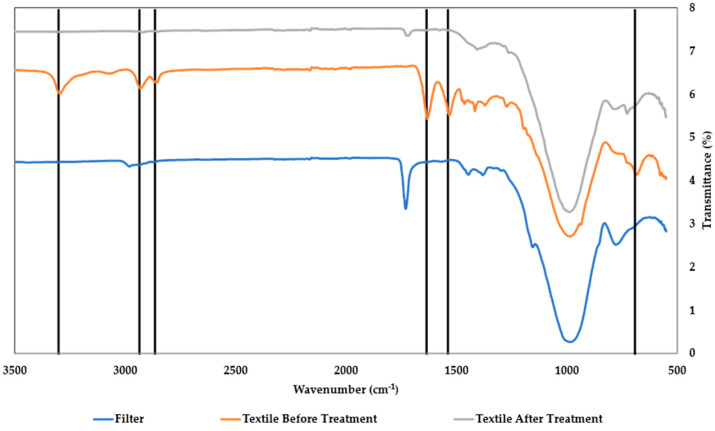
FTIR spectra of the MPs from the textile industry effluent 1, before the in-house WWTP treatment (orange curve) and after (grey curve). The FTIR spectrum of the glass-fibre filter (blue curve) is also shown. The main vibrational modes are highlighted by the solid vertical lines and their assignment is discussed in the main text.

**Figure 9:**
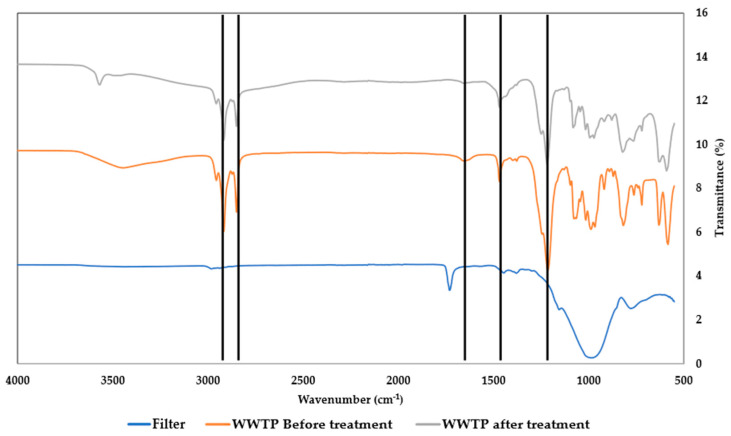
FTIR spectra of the MPs from the municipal WWTP effluent before (orange curve) and after the WWTP (grey curve) and the FTIR spectra from the filter (blue curve). The main vibrational modes are highlighted with the solid vertical lines and their assignment is discussed in the main text.

ATR-FTIR spectroscopy was performed to complement the MP identification, which was initially determined by fluorescence microscopy (Figure 8 and Figure 9).

The FTIR spectra of the MPs recovered from the textile 1 effluent show vibrational modes characteristic of the polyamide network, such as the rocking bands at 1565 cm^−1^ and 1530 cm^−1^, corresponding to the N-H vibration elongation; the band 1265 cm^−1^, assigned to the N-H vibration; and the band 1100 cm^−1^, corresponding to C-O. The band close to 3000 cm^−1^ is assigned to the stretch vibrations of hydrogen atoms, which are bonded as –NH [51]. In addition, the N-H bands confirm the presence of polyamides.

From Figure 9, it is possible to observe the presence of bands at 2880 cm^−1^ and 2950 cm^−1^, attributed to C-H stretching; 1716 cm^−1^, assigned to carbonyl group (C=O) vibration; bands at 1200 cm^−1^ and 1150 cm^−1^, corresponding to asymmetrical C–O–C vibration; and bands at 518 cm^−1^ and 875 cm^−1^, assigned to aromatic rings. These identified vibration modes are characteristic of PET [52,53], thus supporting the data obtained by fluorescence microscopy.

Overall, the data suggest that PET-based MPs are being released to a large extent. These MPs essentially come from textiles originating from the washing of clothes at domestic and industrial levels, a higher contribution from the municipal WWTP effluents having been observed.

**Figure 10:**
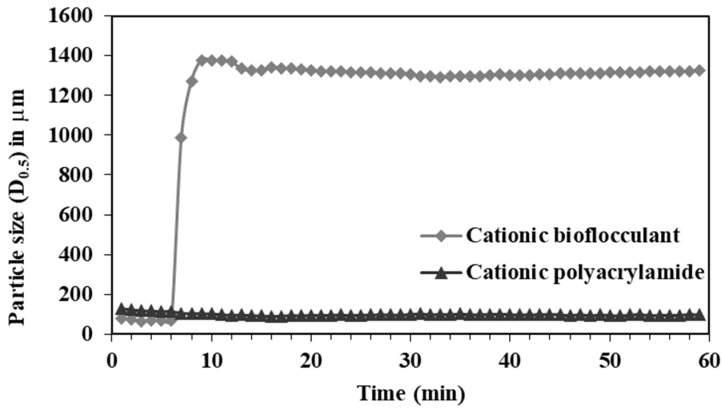
PET flocculation tests using the cationic cellulose derivative coded 048B, with a degree of substitution of 1.02 and a zeta potential of value of +51 mV and a commercial cationic polyacrylamide (supplied by Aqua+Tech, Romont, Switzerland). Evolution of particle size as a function of time, using LDS, with a flocculant concentration of 0.001wt% and using neutral pH values, at room temperature.

Based on the results obtained for the main contaminants in the effluents, the possibility of improving the removal of PET-based MPs using a cationic bio-based flocculant produced from wood wastes was evaluated [54]. The proof-of-concept of the removal process is presented in Figure 10, using a cationic cellulose flocculant and “model” PET MPs, with a concentration, identified in our previous studies as common in effluents, of 0.001 wt% [4], versus a commercial cationic polyacrylamide, of similar molecular weight and at the same concentration.

**Figure 11:**
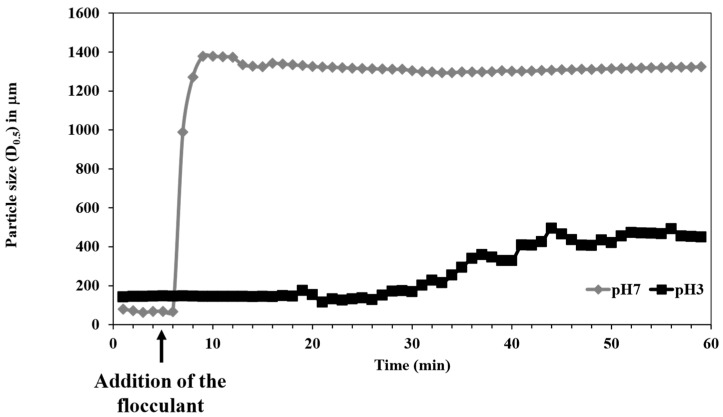
PET flocculation tests using the cationic cellulose derivative coded 048B, with a degree of substitution of 1.02 and a zeta potential of value of +51 mV. Evolution of particle size as function of time, using LDS, with a flocculant concentration of 0.001wt% and using different pH values, at room temperature.

Flocculation experiments conducted under identical conditions revealed a marked difference in performance between the commercial cationic polyacrylamide and the bio-based flocculant. The polyacrylamide showed limited efficiency in promoting flocculation, as no significant change in particle size was observed following its addition. In contrast, the bioflocculant (48B) exhibited superior flocculation efficiency, resulting in a substantial increase in particle size. This indicates a more effective interaction between the bioflocculant and suspended particles, highlighting its potential as a more efficient and environmentally friendly alternative to synthetic flocculants. Additionally, two different pH values were tested to verify the effect of this parameter on the flocculation efficiency, Figure 11.

The flocculation tests performed at pH 3 did not demonstrate any relevant increase in size over time, with only a ca. 300 µm increase observed in the suspended particles after 30 min. Conversely, the flocculation performed at pH 7 resulted in a substantial particle size increase within a few minutes after the addition of the flocculant, demonstrating the great potential of these conditions to aggregate and flocculate MPs and also to remove them, especially those with a high charge density, such as PET. The different effectiveness of the flocculation process is related to the variation in the charge density of the PET MPs at different pHs. The charge density significantly drops with the pH decrease (the zeta potential of PET particles varies from −34.5 to −9.5, at pH 7.0 and 3.0, respectively) due to the protonation of carboxylic groups. This reduction in negative charge on the surface of PET MPs impairs the electrostatic interaction with the quaternary ammonium groups of the cationic bio-based flocculant.

The removal of MPs from municipal and textile wastewater is an important challenge. The present study illustrates some critical aspects including the use of novel cationic cellulose derivatives for the flocculation of MPs. Both the dispersibility and removal of MPs have a basis in particle charge. Typical MPs become increasingly negatively charged as pH is increased from acid to neutral conditions; this is, as mentioned above, due to the ionisation of carboxylic acid groups. It was demonstrated in this study that a cationic polymer causes an efficient removal of MPs in the anionic state but not for less charged particles. Mixed systems of oppositely charged polymers have been an intensive field of research since the pioneering studies of Bungenberg de Jong [55,56,57]. Such literature gives good guidance in opportunities for MP removal by cationic polymers; polymers derived from natural raw materials open a new route.

## 4. Conclusions

In this work, MPs from different textile effluents and a municipal WWTP were extracted, quantified, and characterized. It was found that the treatments applied in the WWTPs (both industrial and municipal) are not efficient enough to completely remove the MPs from the effluents, as a broad range of particle sizes was observed, even in the treated effluents. In the textile 1 effluent, after treatment, a sharp decrease in the median size of particles was noted, suggesting that the effluent treatment generated smaller particles than the native ones. This could be rationalized by the removal of the largest particles and/or by fragmentation induced by the treatments. Likewise, the effluent from the municipal WWTP showed a reduction in particle size between the inlet and outlet effluents. A microscopic observation of the extracted MPs revealed the presence of large particles, suggesting the possible formation of aggregates during treatment. The treatment applied by the municipal WWTP proved to be more efficient than the in-house treatments used in the studied textile industries, removing a larger number of MPs. Both fluorescence microscopy and FTIR-ATR analysis suggest the prevalence of PET-like particles in the effluents, and polyamide in the effluent from the textile industries. The presence of PET in all studied effluents is likely due to its widespread use in packaging and clothing. Finally, when comparing the municipal WWTP effluent with the two textile effluents, the municipal WWTP exhibited a higher quantity of MPs. Most likely, this is because the municipal WWTP receives a wider variety of effluents from different sources, leading to a higher load of MPs at the entrance. The proof-of-concept demonstrates that flocculation with a cationic bio-based polymer can be a suitable approach to efficiently remove MPs from effluents, thereby reducing the pollution caused by these pollutants.

## Figures and Tables

**Table 1:** Zeta potential and pH of effluents, before and after treatment.

	Textile 1		Textile 2		Municipal WWTP	
	Before	After	Before	After	Before	After
pH	13.20	12.89	7.19	6.85	8.00	8.28
Zeta potential (mV)	–16.05 ± 1.08	–25.02 ± 1.11	–28.30 ± 1.47	–31.13 ± 1.40	–13.90 ± 1.10	–12.57 ± 1.52

**Table 2:** Quantification of MPs before and after each WWTP treatment.

Effluents	Before In-House WWTP (mg MPs/100 mL Effluent)	After In-House WWTP (mg MPs/100 mL Effluent)	MPs Released after WWTP (g MPs/Ton Effluent)
Municipal	18.41 ± 2.78	5.42 ± 5.11	54. 20
Textile 1	7.45 ± 1.28	3.36 ± 1.21	33.60
Textile 2	2.11 ± 1.99	1.57 ± 0.21	15.65

## Data Availability

Data are contained within the article or Supplementary Materials.

## Supplementary Materials

The following supporting information can be downloaded at: https://www.mdpi.com/article/10.3390/polym16192803/s1, Figure S1: Two-step cationization of cellulose with GT via periodate oxidation of cellulose to create DAC (1st step), followed by the synthesis of cationic cellulose using Girard’s reagent (2nd step); Figure S2: Oxime reaction between DAC and NH_2_OH.HCl for the quantification of aldehyde groups by elemental analysis; Figure S3: FTIR spectra of cellulose, dialdehyde cellulose and cationized cellulosic materials. Note that the sample 48B is CDAC while the sample coded as DAC_waq_-3 is DAC.
