# Supplementary material for: Insights on Microplastic Contamination from Municipal and Textile Industry Effluents and Their Removal Using a Cellulose-Based Approach
Source: Polymers (Basel). 2024 Oct 3;16(19):2803. doi: 10.3390/polym16192803 (PMC11478531; doi:10.3390/polym16192803)
Supplement: Supplementary file 1 [file polymers-16-02803-s001.zip › polymers-3196241-supplementary.pdf]

Supplementary Information

Insights on Microplastic Contamination from Municipal and Textile Industry Effluents and Their Removal Using a Cellulose-Based Approach

Solange Magalhães, Daniel Paciência, João M. M. Rodrigues, Björn Lindman, Luís Alves, Bruno Medronho and Maria da Graça Rasteiro

Cellulose cationization from dialdehyde cellulose

1st Step:

- Disperse 4 g of cellulose in 250 mL of distilled water.
- Stir the dispersion overnight using a magnetic stirrer.
- Transfer the cellulose suspension to a round flask.
- Dilute the suspension with an additional 200 mL of distilled water.
- Cover the reaction vessel with aluminium foil to prevent the photo-induced decomposition of periodate.
- Place the flask in an oil bath.
- Add 7.2 g of NaIO4 (sodium periodate) and 8.2 g of LiCl (lithium chloride) to the aqueous dispersion, to initiate the oxidation reaction.

Note: LiCl acts as a catalyst and enhances oxidation efficiency by disrupting the hydrogen bonds between cellulose chains, allowing better interaction between the reagents and cellulose.

- Let the reaction proceed for 3 hours.
- After the reaction is complete, filter the product.
- Wash the product several times with distilled water to remove any remaining iodine compounds.

2nd Step:

- Redispersion of DAC in water, for this take 0.8 g of dry DAC (dialdehyde cellulose) and redisperse it in 80 mL of distilled water.
- Add the GT reagent to the mixture. The GT/aldehyde weight ratios should be either 7.8, 3.9, or 1.95 (w/w) depending on the desired cationicity.
- Adjust the pH of the mixture to 4.5 using HCl (hydrochloric acid).
- Allow the reaction to proceed at 70 °C for 1 hour.
- After the reaction is completed, dilute the mixture with isopropanol. This will cause the insoluble products (CDAC) to precipitate out of the solution.
- Centrifuge the precipitated material and wash it with a water/isopropanol mixture (1:9 v/v) to remove any unreacted GT reagent.

The reaction steps and mechanisms are also outlined in Figure SI.1 in the referenced material.

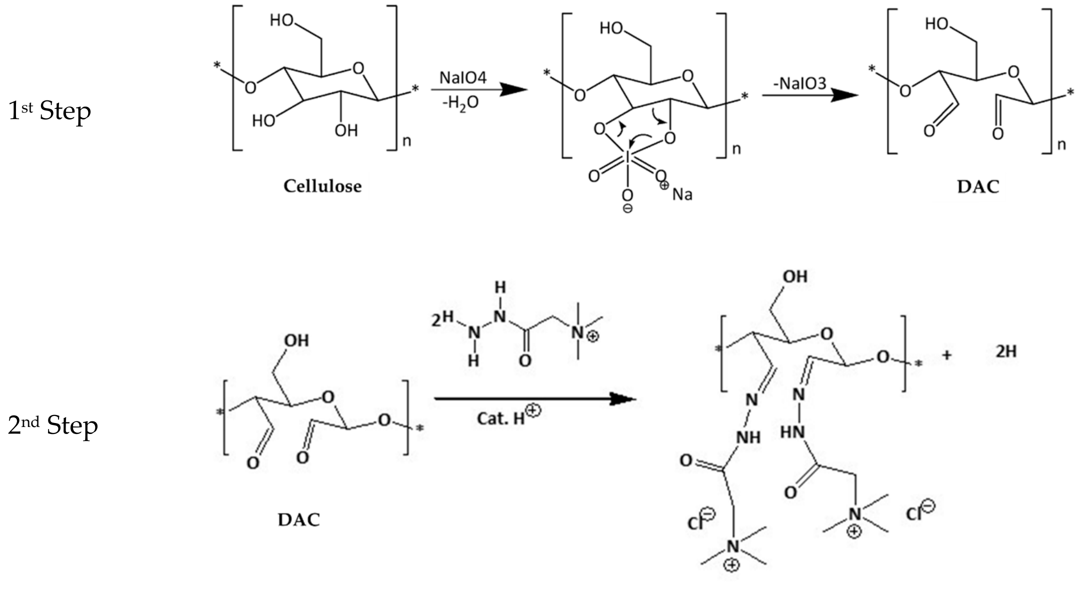

Figure S1: Two-step cationization of cellulose with GT via periodate oxidation of cellulose to create DAC (1st step), followed by the synthesis of cationic cellulose using Girard's reagent (2nd step).

Degree of substitution (DS) of aldehyde groups of the prepared DAC

The determination of the aldehyde content of DAC was based on the oxime reaction (Figure SI.2) between the aldehyde groups of DAC and hydroxylamine hydrochloride ($NH_2OH.HCl$) [1]. 0.1 g of never-dried DAC was placed in a 200 mL beaker containing ca. 1.40 g of NH_2OH. HCl dissolved in 100 mL of 0.1 M acetate buffer (pH = 4.5). The beaker was covered with aluminium foil and the mixture was stirred for 48 h at room temperature. The product was filtrated and washed with deionized water and then dried in a freeze-dryer. The nitrogen content of the obtained product was determined by elemental analysis using an EA 1108 CHNS-O analyser from Fisons.

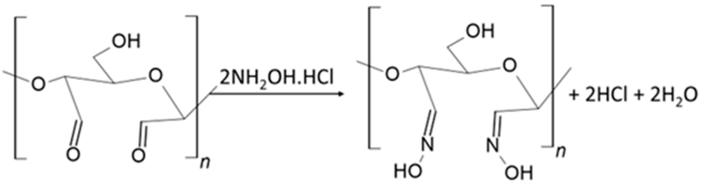

Figure S2: Oxime reaction between DAC and $NH_2OH.HCl$ for the quantification of aldehyde groups by elemental analysis.

Reaction condition for synthesis of cationic wood-based flocculant and characterization results

Name	Time (h)	Temp (ºC)	GT/aldehyde (molar ratio)	DS	Cationicity Index (mmol/g)	ζ-potential (mV)	Z-Av. diameter (nm)	PDI
48B	1	70	1.95	1.02	3.26	51±2	237±18	0.37±0.05

Time= reaction time, DS=degree of substitution, PDI=polydispersity index of the hydrodynamic diameter distribution; dialdehyde cellulose from: DAC, from wood wastes pulp with Kappa N. 13.9.

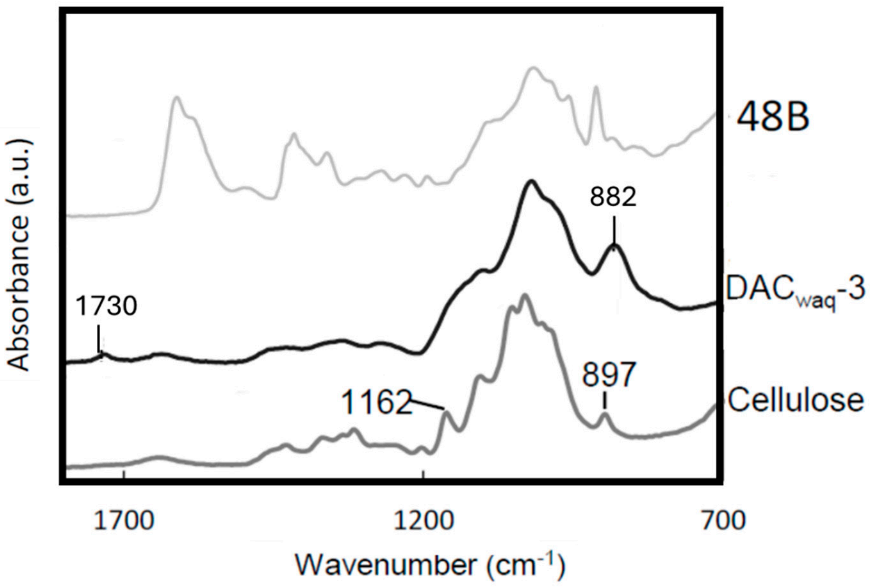

Figure S3: FTIR spectra of cellulose, dialdehyde cellulose and cationized cellulosic materials. Note that the sample 48B is CDAC while the sample coded as DAC_{waq}-3 is DAC.

The FTIR spectra of the starting cellulose material, DAC (dialdehyde cellulose), and CDAC (cationic dialdehyde cellulose) are presented in Figure SI.3. Significant spectral differences were observed between the initial cellulose and the synthesized DAC. In the DAC spectrum, a prominent new absorption band appeared at 1730 cm^{-1}, corresponding to the C=O stretching of aldehyde groups, which was absent in the cellulose spectrum. Additionally, the characteristic C1-H bending band of cellulose at 897 cm^{-1} shifted to 882 cm^{-1} in the DAC spectrum. Changes were also evident in the 1000–1200 cm^{-1} region, particularly with the cellulose band at 1162 cm^{-1}, associated with the asymmetric C-O-C stretching of the glycosidic bond, which became less defined in the DAC spectrum. These alterations can be attributed to ring opening and the oxidation of hydroxyl groups at the C2-C3 positions, with the formation of new hemiacetal linkages between aldehyde and alcohol groups from different chains contributing to these spectroscopic shifts. The obtained results are in agreement with the results reported in the literature [2].

For the CDAC sample (48B), the formation of an imine bond between the oxidised cellulose chains and the Girard T reagent was confirmed by the appearance of a strong band at 1687 cm^{-1}, corresponding to carbonyl stretching in the amide bond (amide I band), and a weaker band at 1559 cm^{-1}, associated with carbon-nitrogen stretching in the amide group (amide II band). Additionally, new bands at 1475 cm^{-1} and 1415 cm^{-1} were observed, corresponding to the asymmetric and symmetric bending of methyl groups, respectively. A sharp band at 925 cm^{-1} was assigned to asymmetric C4-N stretching of the alkylammonium groups, further confirming the successful cationization of DAC to CDAC. The results are aligned with what has been reported in the literature [2].

1. Sirvio, J.; Hyvakko, U.; Liimatainen, H.; Niinimaki, J.; Hormi, O. Periodate oxidation of cellulose at elevated temperatures using metal salts as cellulose activators. *Carbohydr. Polym.* **2011**, *83*, 1293-1297, doi:10.1016/j.carbpol.2010.09.036.
2. Grenda, K.; Gamelas, J.; Arnold, J.; Cayre, O.; Rasteiro, M. Cationization of Eucalyptus wood waste pulps with diverse lignin contents for potential application in colored wastewater treatment. *RSC Adv.* **2019**, *9*, 34814-34826, doi:10.1039/C9RA05757A.
